# Self-reported Depressive Symptoms of school-age siblings of individuals with an Autism Spectrum Disorder (ASD): Findings from a Greek sample

**DOI:** 10.1192/j.eurpsy.2022.1075

**Published:** 2022-09-01

**Authors:** E. Koukouriki

**Affiliations:** University of Ioannina, Special Education Lab, Department Of Primary Education, Ioannina, Greece

**Keywords:** Autism Spectrum Disorders, self-report, depressive symptoms, neurotypical siblings

## Abstract

**Introduction:**

Previous studies have shown elevated levels of depressive symptoms in parents of children with ASD. However, few studies have assessed depressive symptoms in neurotypical ASD-siblings and most have done so, within a broad age range, while studies focusing on a certain developmental stage (middle childhood in particular) using a self-report depression-specific assessment tool are sparse.

**Objectives:**

This study aimed to investigate the depressive symptoms of Greek school-age neurotypical siblings of individuals with an Autism Spectrum Disorder through a self-reported questionnaire.

**Methods:**

The sample included 85 school-age neurotypical ASD-siblings (8-13 years old). The Children’s Depression Inventory (CDI) and a demographics questionnaire were administered to all participants.

**Results:**

Results showed that a considerable percentage of the sample (15.3%) scored twice as high as the mean score. ASD-siblings in the present study scored higher (mean total score in CDI was 7.24±6.27) than children of similar age and nationality. Further, 5.9% of the siblings in the present study exhibited severe depressive symptoms (using 19 as a threshold) whereas 12.9% of ASD-siblings scored above 15 and therefore should be further evaluated by mental health services.

**Conclusions:**

The results of the present study documents a relatively high prevalence of depressive symptoms in neurotypical siblings of individuals with ASD. ASD-siblings showed higher levels of depressive symptoms compared to normative data. This is the first study addressing depressive symptoms in siblings of autistic children conducted in the Greek cultural context. The present study highlights the need for the development and implementation of appropriate and effective interventions within the Greek healthcare system for ASD-siblings.

**Disclosure:**

No significant relationships.

